# *SDC1* and *ITGA2* as novel prognostic biomarkers for PDAC related to IPMN

**DOI:** 10.1038/s41598-023-44646-x

**Published:** 2023-10-31

**Authors:** Chuan-long Zhang, Qian Shen, Fu-dong Liu, Fan Yang, Meng-qi Gao, Xiao-chen Jiang, Yi Li, Xi-yuan Zhang, Ge-er En, Xue Pan, Bo Pang

**Affiliations:** 1 Guang’anmen Hospital, China Academy of Chinese Medical Sciences, Beijing, 100053 China; 2 Beijing University of Chinese Medicine, Beijing, 100029 China; 3 Wangjing Hospital, China Academy of Chinese Medical Sciences, Beijing, 100102 China; 4 Third Affiliated Hospital, Beijing University of Chinese Medicine, Beijing, 100029 China; 5 International Medical Department of Guang’anmen Hospital China Academy of Chinese Medical Sciences, Beijing, 100053 China

**Keywords:** Biotechnology, Cancer, Genetics, Immunology, Biomarkers, Molecular medicine

## Abstract

The existing biomarkers are insufficient for predicting the prognosis of pancreatic ductal adenocarcinoma (PDAC). Intraductal papillary mucinous neoplasm (IPMN) is a precursor to PDAC; therefore, identifying biomarkers from differentially expressed genes (DEGs) of PDAC and IPMN is a new and reliable strategy for predicting the prognosis of PDAC. In this study, four datasets were downloaded from the Gene Expression Omnibus database and standardized using the R package ‘limma.’ A total of 51 IPMN and 81 PDAC samples were analyzed, and 341 DEGs in PDAC and IPMN were identified; DEGs were involved in the extracellular matrix and tumor microenvironment. An acceptable survival prognosis was demonstrated by *SDC1* and *ITGA2*, which were highly expressed during in vitro PDAC cell proliferation, apoptosis, and migration. *SDC1*^high^ was enriched in interferon alpha (IFN-α) response and *ITGA2*^high^ was primarily detected in epithelial-mesenchymal transition (EMT), which was verified using western blotting. We concluded that *SDC1* and *ITGA2* are potential prognostic biomarkers for PDAC associated with IPMN. Downregulation of *SDC1* and *ITGA2* expression in PDAC occurs via a mechanism involving possible regulation of IFN-α response, EMT, and immunity, which may act as new targets for PDAC therapy.

## Introduction

Pancreatic cancer (PC) is a type of malignant tumor with one of the highest mortality rates. According to the latest epidemiological data, 64,050 new cases of PC and 50,550 new cancer-related deaths have been recorded worldwide in 2023^1^. Pancreatic ductal adenocarcinoma (PDAC) is the most common type of PC^2^. The 5-year survival rate of patients with PDAC is less than 10%^3^. Development of an effective treatment for PC has become a major clinical challenge. For traditional treatments, such as radiotherapy and chemotherapy, early (or late) drug resistance in patients with PDAC is always a concern^4^. Similarly, immunotherapy is being increasingly used for PDAC treatment, but significant challenges have been encountered because of the special tumor microenvironment (TME), which makes it difficult for immune cells to infiltrate and activate^5^. The identification of novel prognostic biomarkers will provide a strategy for the prevention of PDAC and identification of newer therapeutic targets.

The forward shift of the treatment threshold for PDAC is currently advocated as an effective and prudent move, and effective prognostic markers can demonstrate the clinical value in disease prevention. With the rapid development of imaging technology, the diagnosis of precancerous lesions associated with PC has become easier, which has focused research efforts on these precancerous lesions. Intraductal papillary mucinous neoplasm (IPMN), a precancerous lesion, is considered the most common type of pancreatic cystic tumor^6^. First discovered and described by Ohashi in 1982, it is the second most common pancreatic tumor^7^. IPMN is a rare tumor that originates from the epithelium^8^. It is a mucin-producing tumor that secretes a large amount of mucus and forms pancreatic cysts, which can gradually become malignant through the ‘hyperplasia-adenoma-cancer’ pathway^9,10^. There are three main types of IPMN: the main pancreatic duct type (MD-IPMN), branch-duct type (BD-IPMN), and mixed type^11^. Among them, the incidence of malignancy in MD-IPMN ranges from 57–92%^12^. Before IPMN develops into PDAC, there must be differences in gene expression between tissues.

The development of bioinformatics analysis and public databases^13,14^ can facilitate an understanding of the molecular mechanisms underlying IPMN and PDAC. The data of these platforms provide support for searching effective prognostic biomarkers and therapeutic targets. COL10A1, mediated by noncoding RNA, indicates that the prognosis of PC is poor. It involves mechanisms related to immunity^15^. In addition, we previously established seven immune-related miRNAs, including TNFSF9, TNFRSF9, KIR3DL1, and HAVCR2, as better prognostic models for PDAC^16^. Wang et al.^17^ studied miRNAs in the cystic fluid of low-grade benign and highly invasive pancreatic cystic lesions by sequencing and identified that miRNAs, such as miR-216a and miR-217, may be early detection biomarkers for pancreatic cystic lesions developing into PDAC. Early PDAC detection is associated with better survival^18^. Given the difficulty of diagnosis and treatment of PDAC, the identification of PDAC prognostic markers from precancerous and non-invasive lesions will be a reliable strategy; bioinformatics analysis makes this identification easier. The differentially expressed genes (DEGs) between IPMN and PDAC can be identified using bioinformatics programs^19,20^. New prognostic biomarkers are identified to determine whether they have predictive value for the survival of PDAC. Thus, identifying survival-related biomarkers of PDAC from these DEGs is a reliable strategy for establishing new prognostic biomarkers.

To identify potential biomarkers and predict the prognosis of patients with PDAC, we conducted bioinformatics analyses to identify DEGs between IPMN and PDAC samples. We then analyzed the DEGs using enrichment analysis to explore the possible mechanisms underlying the transformation from IPMN to PDAC. We further screened hub genes using bioinformatics methods. A survival analysis of the hub genes was performed to screen out the survival-related biomarkers for PDAC. We studied their clinical value based on public data. Furthermore, we verified their effects on the biological behavior of PDAC cells in vitro. Then, we performed the preliminary exploration and verification of the internal mechanisms. Our results indicated that *SDC1* and *ITGA2* are potential prognostic biomarkers of PDAC.

## Results

### Identification of DEGs

**Figure 1:**
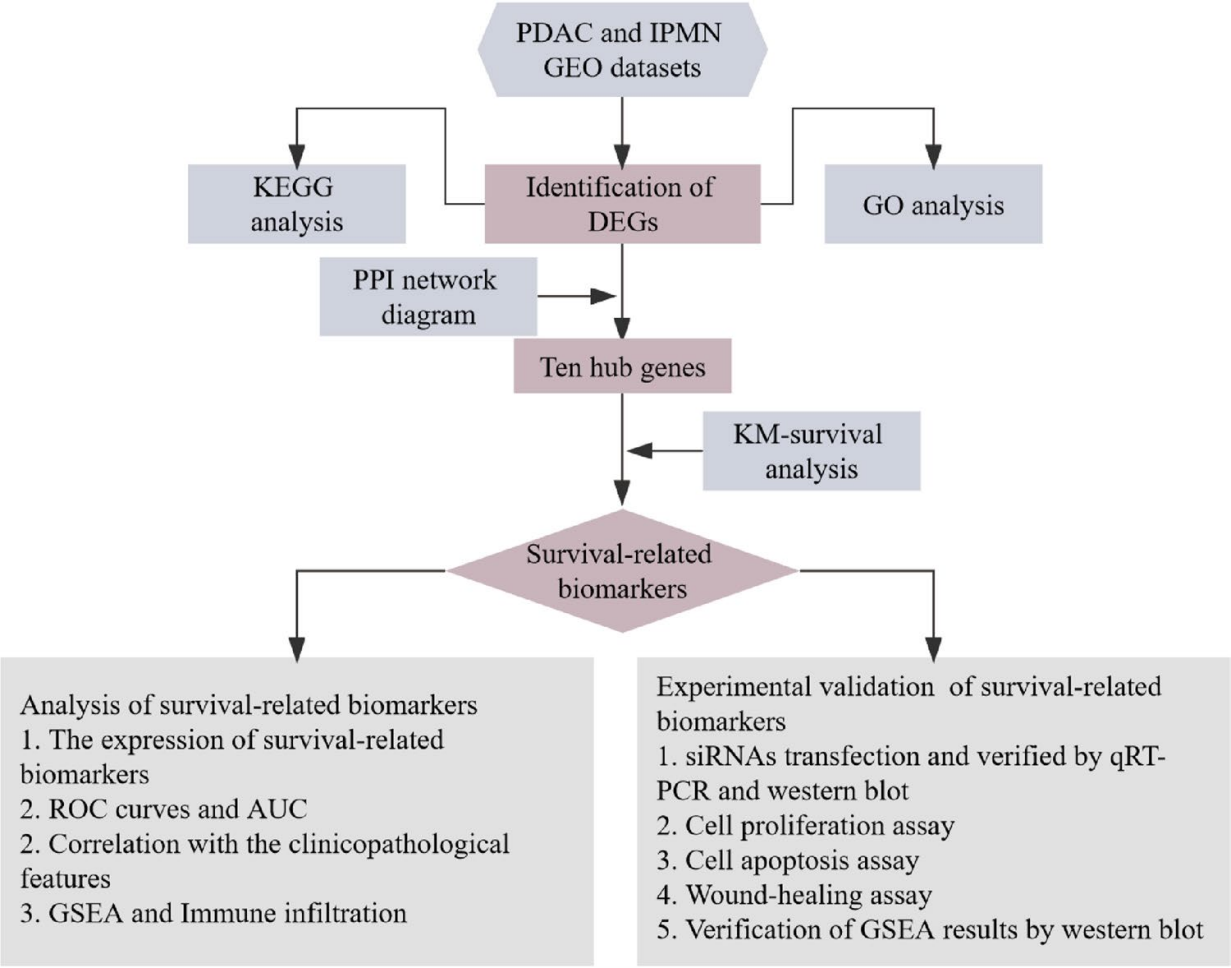
Flow chart of biomarker identification and validation. *PDAC* Pancreatic ductal adenocarcinoma; *IPMN* Intraductal papillary mucinous neoplasm; *GEO DEGs* Differentially expressed genes; *GO* Gene Ontology; *KEGG* Kyoto Encyclopedia of Genes and Genomes; *PPI* Protein–protein interaction; *K–M* Kaplan–Meier; *GSEA* Gene set enrichment analysis; *ROC* Receiver operating characteristics; *AUC* Area under the curve; *qRT-PCR* Quantitative real-time polymerase chain reaction.

**Figure 2:**
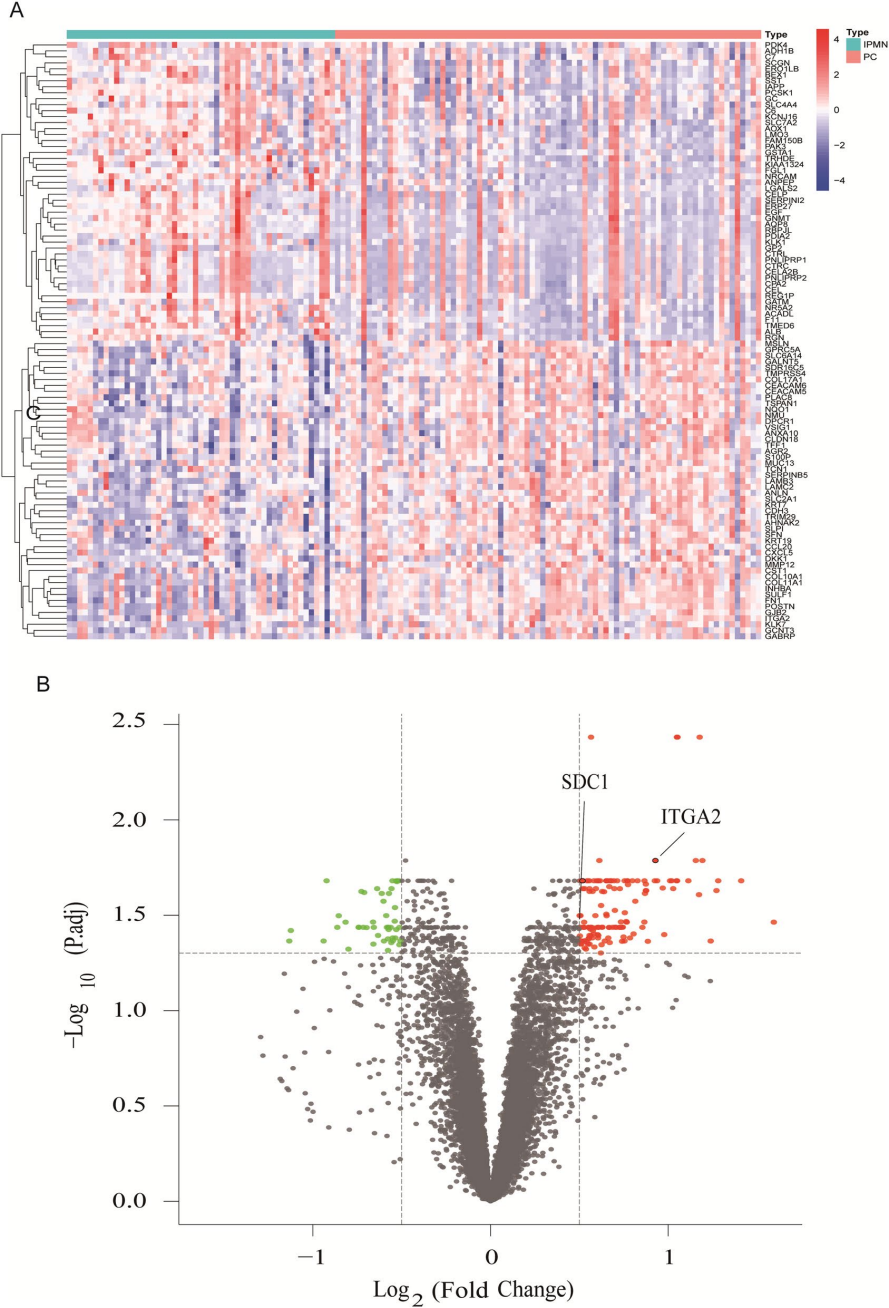
Identification of DEGs between PDAC and IPMN. (**A**) The heat map illustrates the differential expression of 341 DEGs between IPMN and PDAC samples identified with the thresholds of |log_2_ fold-change|> 0.5 and adjusted *P*-value < 0.05. (**B**) Volcano plots of DEGs. Colors represent the expression level of the genes, as follows: red, upregulated; green, downregulated; and gray, not statistically significant. *PDAC* Pancreatic ductal adenocarcinoma; *IPMN* Intraductal papillary mucinous neoplasm; *DEGs* Differentially expressed genes.

The flowchart is shown in Fig. 1. A total of 132 samples (51 IPMN and 81 PDAC) were included in this study. In total, 341 DEGs were identified (Fig. 2A). As shown in Fig. 2B, 230 genes were upregulated, and 111 were downregulated.

### Enrichment analysis results

**Figure 3:**
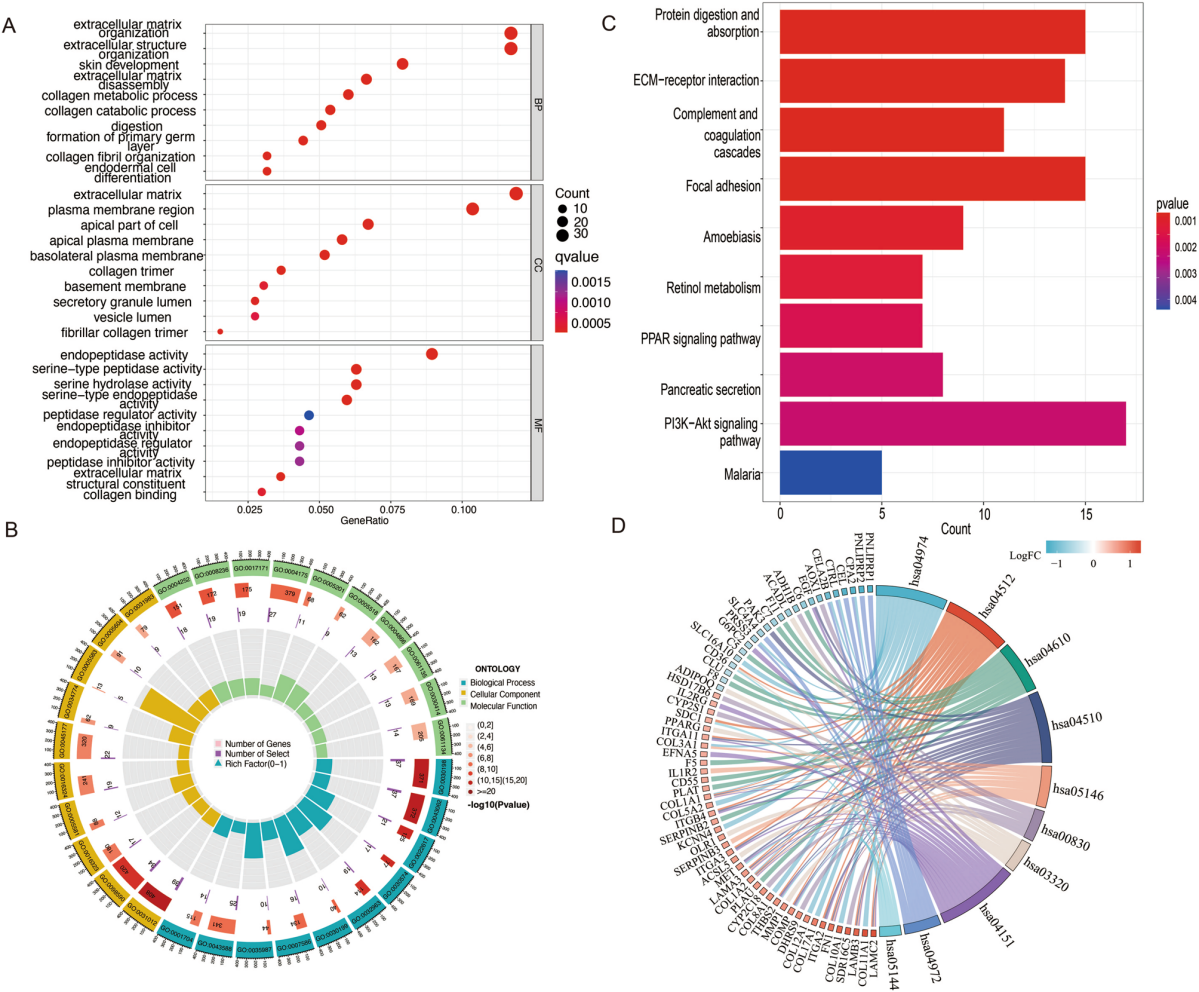
Functional enrichment analysis. (**A**) GO functional enrichment analysis of the 341 DEGs, the most significant one is marked in red. (**B**) GO Circle plot; blue represents biological process (BP), yellow represents cellular component (CC), and green represents molecular function (MF). (**C**) KEGG functional enrichment analysis of the 341 DEGs, the most significant one is marked in red. (**D**) KEGG Chord plot; the size of the arc represents the correlation between the pathway and pancreatic cancer. Abbreviations: *DEGs* Differentially expressed genes; *GO* Gene Ontology; *KEGG* Kyoto Encyclopedia of Genes and Genomes.

The results of the GO analysis are shown in Fig. 3A and B. The extracellular matrix (ECM) was particularly emphasized. KEGG analysis showed that these DEGs are mainly involved in pathways such as the PI3K-Akt signaling pathway, protein digestion and absorption, complement and coagulation cascades, focal adhesion, amoebiasis, retinol metabolism, and pancreatic secretion (Fig. 3C,D).

### Identification of hub genes

**Figure 4:**
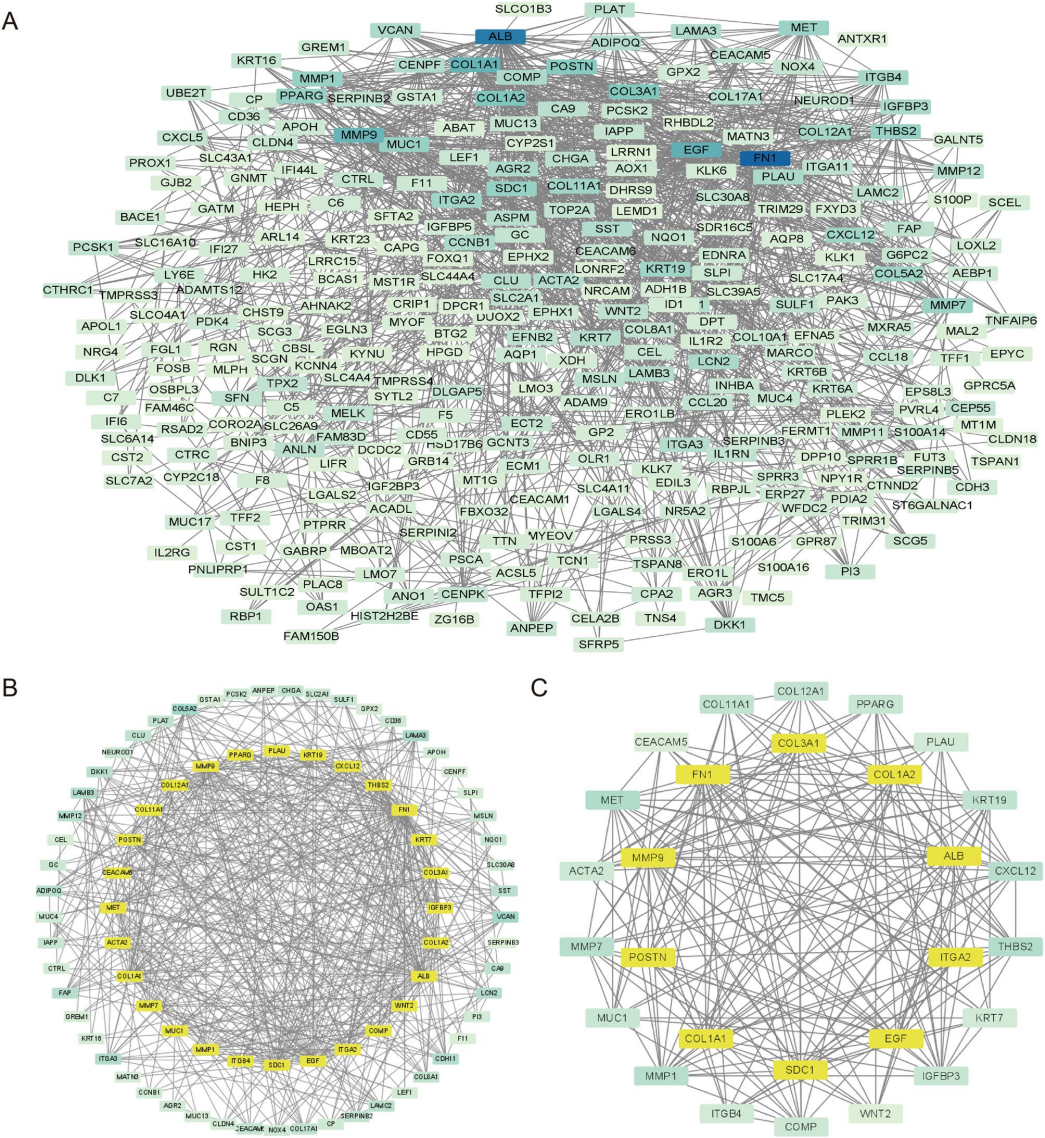
Identification of hub genes through a PPI network diagram. (**A**) PPI network of 341 DEGs. Different node colors were determined based on the logFC of DEGs; the higher the degree, the darker the color. (**B**) Results of the cytoHubba topological analysis. (**C**) Hub genes network module diagram. The top ten hub genes sorted by degree are marked in yellow. Abbreviations: *PPI* Protein–protein interaction; *DEGs* Differentially expressed genes.

The constructed PPI network consisted of 298 nodes and 1250 edges (Fig. 4A). The top 10 genes were screened using the “CytoHubba” (plugin). The interaction mode between DEGs was determined based on key genes (Fig. 4B,C). The gene and protein names and the degrees of these hub genes are listed in Table S1.

### SDC1 and ITGA2 are potential prognostic biomarkers of PDAC related to IPMN

**Figure 5:**
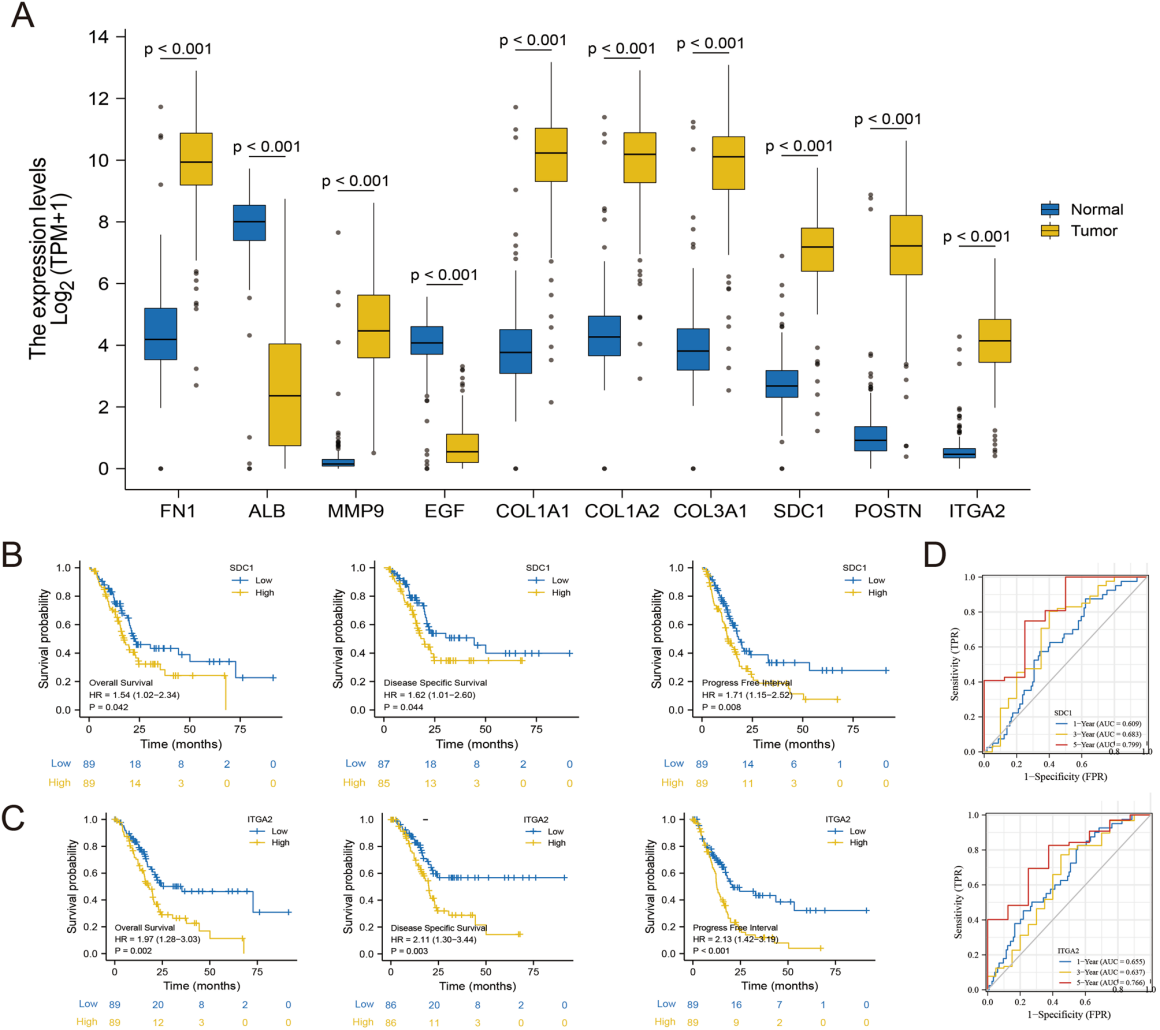
Selection of survival-related biomarkers. (**A**) Ten hub gene expression differences in pancreatic samples of the TCGA and GTEx datasets (171 normal samples and 179 pancreatic cancer samples). (**B**, **C**) K‑M curves and Cox regression between the high-risk and low-risk groups of *SDC1* and *ITGA2* in the TCGA-PAAD dataset. (**D**) Time-dependent ROC curve analysis measuring the predictive performance on 1-, 3-, and 5-year OS of *SDC1* and *ITGA2* in the TCGA-PAAD dataset. *K–M* Kaplan–Meier; *OS* Overall survival; *ROC* Receiver operating characteristic; *AUC* Area under the curve.

Among the top 10 hub genes, *FN1*, *MMP9*, *COL1A1*, *COL1A2*, *COL3A1*, *SDC1*, *POSTN*, and *ITGA2* were highly expressed in PC tissues (Fig. 5A). The results showed that high expression of *SDC1 (SDC1*^high^) significantly affected the survival of patients with PDAC (HR_OS_: 1.54, 95% CI 1.02–2.34, *P* = 0.042; HR_DSS_: 1.62, 95% CI 1.01–2.60, *P* = 0.044; HR_PFI_: 1.71, 95% CI 1.15–2.52, *P* = 0.008; Fig. 5B). Similarly, the high expression of *ITGA2 (ITGA2*^high^) can also be a good predictor of poor prognosis in patients with PDAC. (HR_OS_: 1.97, 95% CI 1.28–3.03, *P* = 0.002; HR_DSS_: 2.11, 95% CI 1.30–3.44, *P* = 0.003; HR_PFI_: 2.13, 95% CI 1.42–3.19, *P* < 0.001; Fig. 5C). The K–M curves of the other genes are shown in Supplementary Figure S1.

As shown by the ROC curves, *SDC1* and *ITGA2* had an acceptable predictive effect on survival prognosis, especially in the prediction of a 5-year survival rate. For *SDC1*^high^, the AUC_1-year_ was 0.609, AUC_3-year_ was 0.683, and AUC_5-year_ was 0.799. For *ITGA2*^high^, the AUC_1-year_ was 0.655, AUC_3-year_ was 0.637, and AUC_5-year_ was 0.766 (Fig. 5D).

Based on these results, 178 patients of the TCGA-PAAD dataset were equally divided into two groups. The cutoff value of the division is the median expression level of *SDC1* and *ITGA2*. In addition, we analyzed the clinicopathological characteristics of the patients according to the high and low expression of *SDC1* and *ITGA2* (Table 1**)**. The expression of *SDC1* and *ITGA2* were affected by ‘primary therapy outcome’ and ‘histologic grade’ (*P* < 0.05). There were also significant differences in *ITGA2*^high^ and *ITGA2*^low^ between different ‘T stages’ and ‘pathologic stages’ (*P* < 0.05). Other clinicopathological characteristics were not affected.

**Table 1:** Correlation between *SDC1* and *ITGA2* and clinicopathological characteristics of patients with PC.

Characteristic	Level	Low expression of *SDC1*	High expression of *SDC1*	*P*	Low expression of *ITGA2*	High expression of *ITGA2*	*P*
N		89	89		89	89	
T stage, n (%)	T1	5 (2.8%)	2 (1.1%)	0.435	4 (2.3%)	3 (1.7%)	**0.024**
	T2	14 (8%)	10 (5.7%)		18 (10.2%)	6 (3.4%)	
	T3	66 (37.5%)	76 (43.2%)		63 (35.8%)	79 (44.9%)	
	T4	2 (1.1%)	1 (0.6%)		2 (1.1%)	1 (0.6%)	
N stage, n (%)	N0	25 (14.5%)	25 (14.5%)	1.000	27 (15.6%)	23 (13.3%)	0.517
	N1	60 (34.7%)	63 (36.4%)		58 (33.5%)	65 (37.6%)	
M stage, n (%)	M0	42 (50%)	37 (44%)	0.055	34 (40.5%)	45 (53.6%)	0.396
	M1	0 (0%)	5 (6%)		1 (1.2%)	4 (4.8%)	
Pathologic stage, n (%)	Stage I	13 (7.4%)	8 (4.6%)	0.067	16 (9.1%)	5 (2.9%)	**0.017**
	Stage II	72 (41.1%)	74 (42.3%)		67 (38.3%)	79 (45.1%)	
	Stage III	2 (1.1%)	1 (0.6%)		2 (1.1%)	1 (0.6%)	
	Stage IV	0 (0%)	5 (2.9%)		1 (0.6%)	4 (2.3%)	
Primary therapy outcome, n (%)	PD	16 (11.5%)	33 (23.7%)	**0.008**	11 (7.9%)	38 (27.3%)	** < 0.001**
	SD	4 (2.9%)	5 (3.6%)		5 (3.6%)	4 (2.9%)	
	PR	7 (5%)	3 (2.2%)		5 (3.6%)	5 (3.6%)	
	CR	44 (31.7%)	27 (19.4%)		42 (30.2%)	29 (20.9%)	
Gender, n (%)	Female	41 (23%)	39 (21.9%)	0.880	40 (22.5%)	40 (22.5%)	1.000
	Male	48 (27%)	50 (28.1%)		49 (27.5%)	49 (27.5%)	
Race, n (%)	Asian	4 (2.3%)	7 (4%)	0.685	5 (2.9%)	6 (3.4%)	0.925
	Black or African American	3 (1.7%)	3 (1.7%)		3 (1.7%)	3 (1.7%)	
	White	79 (45.4%)	78 (44.8%)		81 (46.6%)	76 (43.7%)	
Residual tumor, n (%)	R0	57 (34.8%)	50 (30.5%)	0.360	57 (34.8%)	50 (30.5%)	0.180
	R1	26 (15.9%)	26 (15.9%)		20 (12.2%)	32 (19.5%)	
	R2	1 (0.6%)	4 (2.4%)		3 (1.8%)	2 (1.2%)	
Histologic grade, n (%)	G1	25 (14.2%)	6 (3.4%)	** < 0.001**	23 (13.1%)	8 (4.5%)	**0.003**
	G2	44 (25%)	51 (29%)		44 (25%)	51 (29%)	
	G3	17 (9.7%)	31 (17.6%)		18 (10.2%)	30 (17%)	
	G4	2 (1.1%)	0 (0%)		2 (1.1%)	0 (0%)	
Smoker, n (%)	No	33 (22.9%)	32 (22.2%)	0.650	36 (25%)	29 (20.1%)	0.581
	Yes	36 (25%)	43 (29.9%)		39 (27.1%)	40 (27.8%)	
History of diabetes, n (%)	No	48 (32.9%)	60 (41.1%)	0.337	50 (34.2%)	58 (39.7%)	0.298
	Yes	21 (14.4%)	17 (11.6%)		22 (15.1%)	16 (11%)	
History of chronic pancreatitis, n (%)	No	63 (44.7%)	65 (46.1%)	0.132	63 (44.7%)	65 (46.1%)	1.000
	Yes	3 (2.1%)	10 (7.1%)		6 (4.3%)	7 (5%)	
Family history of cancer, n (%)	No	20 (18.2%)	27 (24.5%)	0.321	24 (21.8%)	23 (20.9%)	0.544
	Yes	34 (30.9%)	29 (26.4%)		37 (33.6%)	26 (23.6%)	
Alcohol history, n (%)	No	36 (21.7%)	29 (17.5%)	0.281	27 (16.3%)	38 (22.9%)	0.112
	Yes	46 (27.7%)	55 (33.1%)		56 (33.7%)	45 (27.1%)	
Age, mean ± SD		63.98 ± 10.86	65.52 ± 10.74	0.343	65.64 ± 10.03	63.85 ± 11.5	0.271

Significant values are in bold.

### Depletion of SDC1 and ITGA2 suppressed proliferation, induced apoptosis, and impaired migration in PDAC cells in vitro

**Figure 6:**
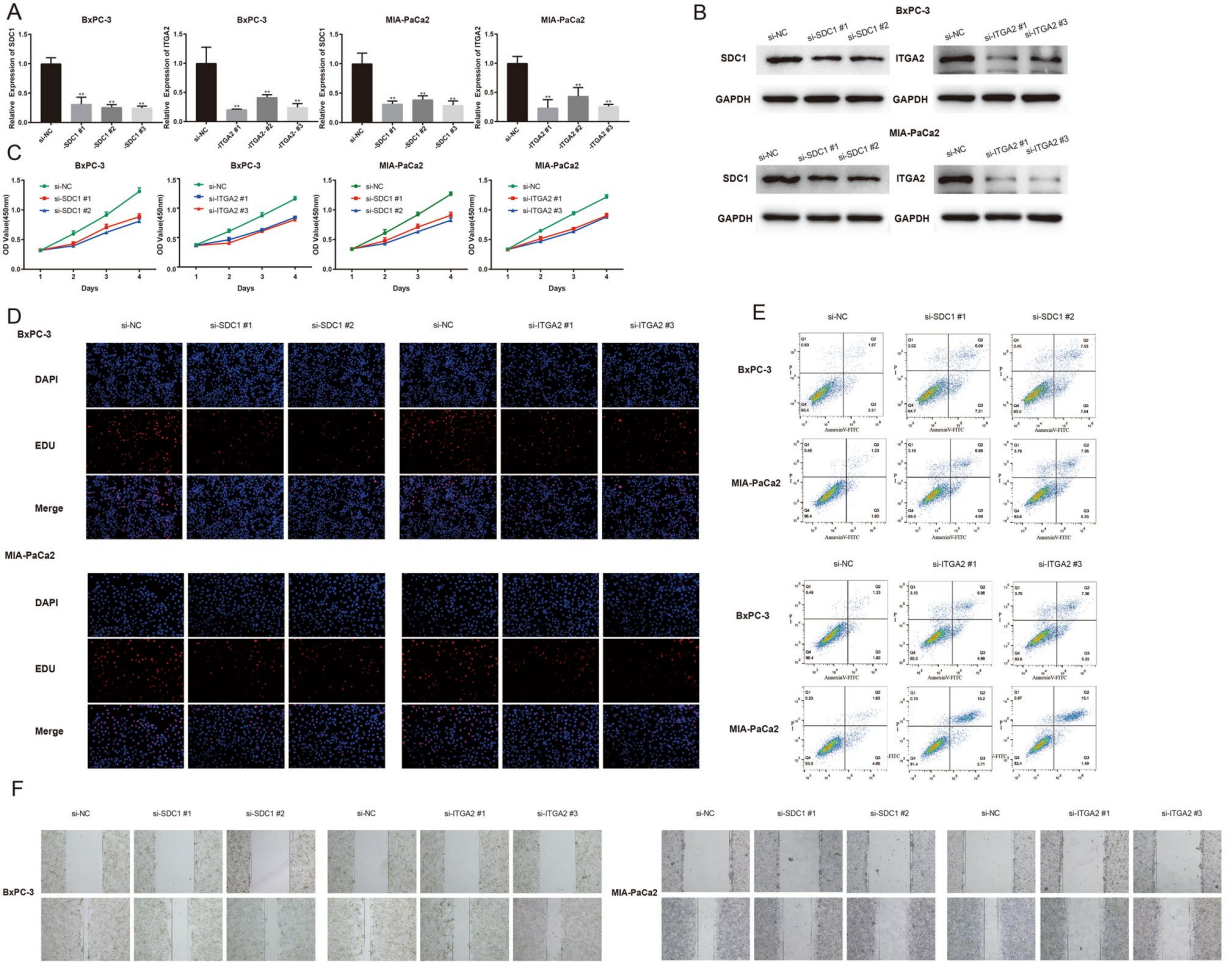
SiRNA-mediated knockdown of *SDC1* and *ITGA2* regulates PDAC cell proliferation, apoptosis, and invasion in vitro. (**A**) BxPC-3 and MIA-PaCa2 were transfected with siRNAs. qRT-PCR was used to detect the transfection efficiency. (**B**) Si-SDC1 #1, si-SDC1 #2, si-ITGA2 #1, and si-ITGA2 #3 had better knockdown effects and were selected for further studies, which were detected by western blot. (**C**, **D**) CCK-8 assay and EdU assay were conducted to examine the BxPC-3 and MIA-PaCa2 cell proliferation viability after the knockdown of *SDC1* and *ITGA2*. (**E**) Knocking down *SDC1* and *ITGA2* expression promoted apoptosis in PDAC cells. (**F**) Migration of BxPC-3 and MIA-PaCa2 was detected after transfection with si-SDC1 and si-ITGA2, respectively, for 0 h and 24 h. These data are representative of three independent experiments with similar results. *PDAC* Pancreatic ductal adenocarcinoma; *qRT-PCR* Quantitative real-time polymerase chain reaction; *CCK-8* Cell Counting Kit-8. (ns *P* ≥ 0.05; *P < 0.05; **P < 0.01; ***P < 0.001; ****P < 0.0001).

We further elucidated the role of *SDC1* and *ITGA2* in the biological behavior of PDAC cell lines. *SDC1* and *ITGA2* were knocked down in PDAC cells using siRNA targeting the gene sequence. Transfection efficiency was examined using qRT-PCR (Fig. 6A) and western blotting (Fig. 6B**)**. Si-SDC1 #1, si-SDC1 #2, si-ITGA2 #1, and si-ITGA2 #3 showed the best knockdown effects and were selected for further studies. The proliferative capacities of PDAC cells were remarkably suppressed when *SDC1* and *ITGA2* were downregulated, as illustrated by the CCK-8 assay (Fig. 6C). The results of the EdU assay were similar (Fig. 6D and Figure S2). Knockdown of *SDC1* and *ITGA2* promoted apoptosis in PDAC cells (Fig. 6E). Next, we found that PDAC cell migration was impaired after *SDC1* and *ITGA2* knockdown (Fig. 6F).

### GSEA and immune cell infiltration results

**Figure 7:**
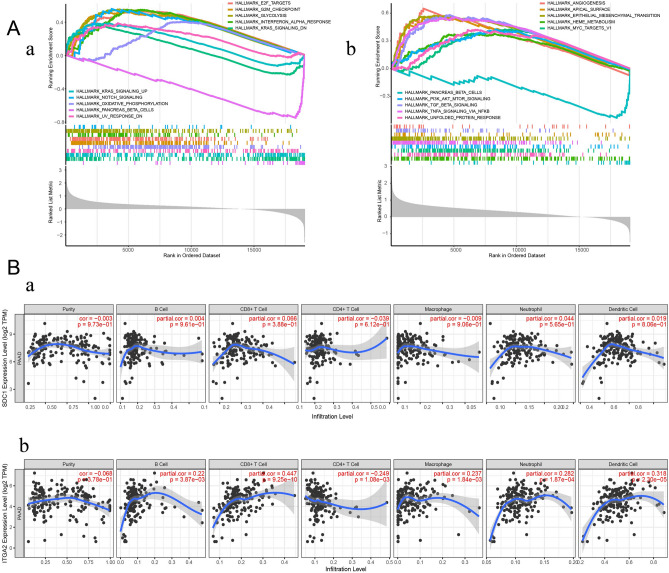
Results of GSEA and immune cell infiltration. (**A**) The results of GSEA analysis. (**a**) For *SDC1* (**b**) For *ITGA*2. (**B**) Correlation analysis between two survival-related biomarkers and tumor-infiltrating immune cells based on the TIMER web tool. *P*-value as indicated. (**a**) For *SDC1* (**b**) For *ITGA2*.

The GSEA results showed that *SDC1*^high^ was mainly found in E2F targets, G2M checkpoint, glycolysis, IFN-α response, and KRAS signaling in DN. *ITGA2*^high^ was mainly detected in angiogenesis, apical surface, epithelial–mesenchymal transition (EMT), heme metabolism, and MYC targeting the V1 pathway. Both *SDC1*^low^ and *ITGA2*^low^ were enriched in the pancreatic β-cell pathway (Fig. 7A).

There were no significant immune correlations with *SDC1*. A positive correlation was identified for *ITGA2* expression and the levels of B cells (*P* = 3.87 × 10^−3^), CD8^+^ T cells (*P* = 9.25 × 10^−10^), macrophages (*P* = 1.84 × 10^−3^), neutrophils (*P* = 1.87 × 10^−4^), and dendritic cells (*P* = 2.30 × 10^−5^), indicating the key role of *ITGA2* expression in immune infiltration (Fig. 7B).

### Depletion of SDC1 and ITGA2 suppressed EMT, enhanced the expression of pro-inflammatory cytokines, and promoted tumor growth

**Figure 8:**
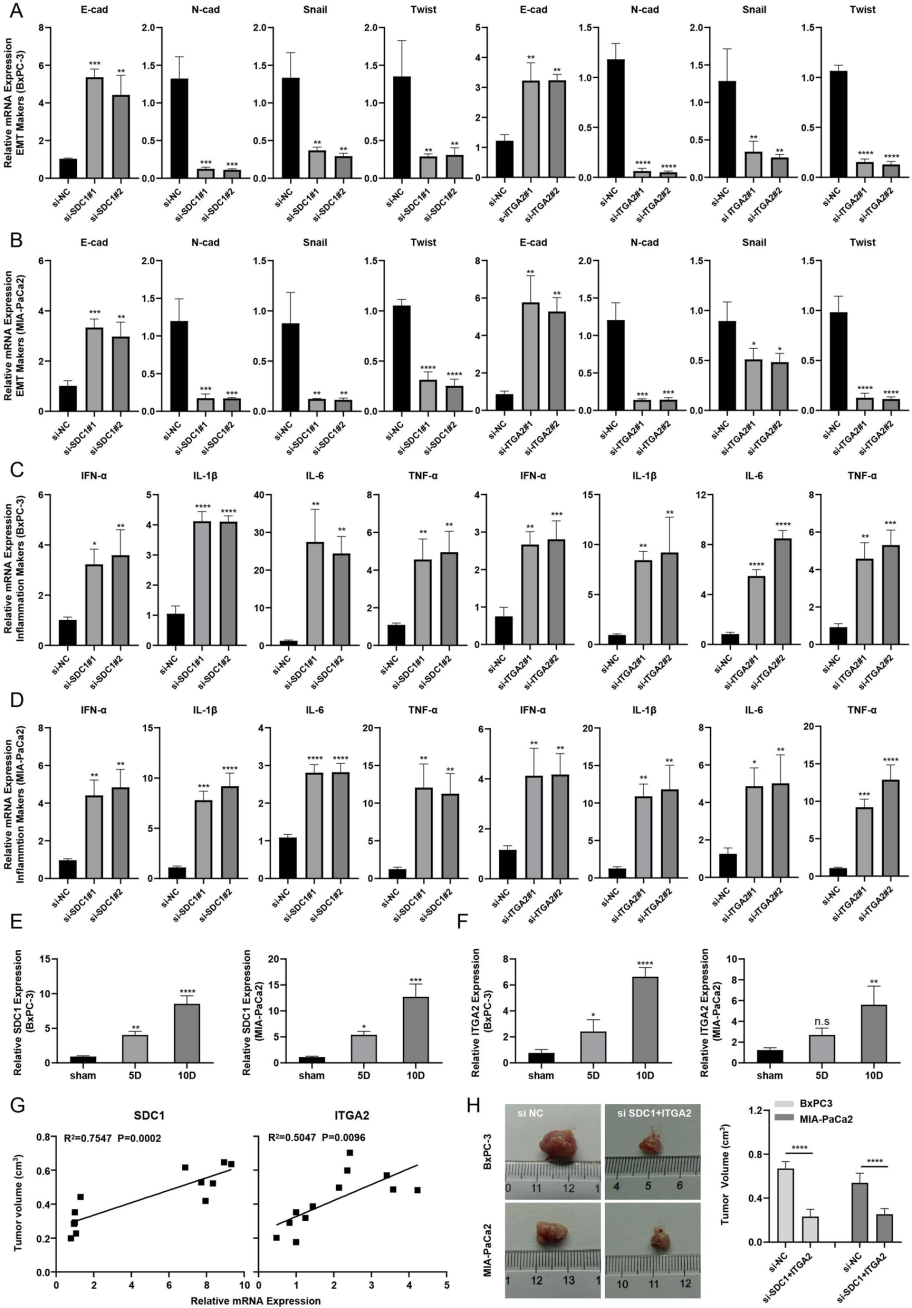
Depletion of *SDC1* and *ITGA2* suppressed EMT, enhanced the expression of pro-inflammatory cytokines, and promoted tumor growth. (**A**) Si-SDC1 and si-ITGA2 inhibited EMT in BxPC-3 cells, as indicated by qRT-PCR; (**B**) Si-SDC1 and si-ITGA2 inhibited EMT in MIA-PaCa2 cells, as indicated by qRT-PCR; (**C**) Si-SDC1 and si-ITGA2 upregulated the expression of pro-inflammatory cytokines in BxPC-3 cells; (**D**) Si-SDC1 and si-ITGA2 upregulated the expression of pro-inflammatory cytokines in MIA-PaCa2 cells; (**E**) Relative *SDC1* expression in tumor-bearing nude mice; (**F**) Relative *ITGA2* expression in tumor-bearing nude mice; (**G**) Regression curves of relative mRNA expression and tumor volume; (**H**) Knocking down of *SDC1* and *ITGA2* inhibited tumor growth. Abbreviations: *EMT* Epithelial–mesenchymal transition; *qRT-PCR* Quantitative real-time polymerase chain reaction. (ns *P* ≥ 0.05; *P < 0.05; **P < 0.01; ***P < 0.001; ****P < 0.0001).

We used qRT-PCR to detect changes in EMT makers and cytokine makers in BxPC-3 and MIA-PaCa2 cell lines. The results showed that after inhibiting the expression of *SDC1* and *ITGA2,* the EMT process of pancreatic cancer cell lines was inhibited; this was manifested by an increased expression of E-cadherin and decreased expression of N-cadherin, Snail, and Twist (Fig. 8A,B). Subsequently, we observed that after inhibiting the expression of *SDC1* and *ITGA2*, the expression of pro-inflammatory cytokines was upregulated in BxPC-3 and MIA-PaCa2 cell lines, shown by increased transcription levels of IFN-α, IL-1β, IL-6 and TNF-α (Fig. 8C,D).

Thereafter, we inoculated BxPC-3 and MIA-PaCa2 cell lines into immunodeficient mice to construct a mouse tumorigenic model, and detected the transcription levels of *SDC1* and *ITGA2*, by qRT-PCR, in tumor tissues on the fifth and tenth days after tumorigenesis by qRT-PCR (Fig. 8E,F). The results showed that with the extension of inoculation time of pancreatic cancer cells, the expression levels of *SDC1* and *ITGA2* in tumor tissues also increased. Regression analysis of tumor tissue volume and expression of *SDC1* and *ITGA2* showed a positive correlation ed with the expression of *SDC1* and *ITGA2* (Fig. 8G, Table S4,S4). Finally, we found that after inhibiting *SDC1* and *ITGA2* expression in BxPC-3 and MIA-PaCa2 cell lines, the tumor size was significantly reduced, showing a potential therapeutic effect (Fig. 8H, Table S5,S5).

## Discussion

The current survival rate of patients with PDAC is not ideal. Owing to the lack of effective means to predict the prognosis, it is difficult for doctors and patients to choose the best treatment when PDAC occurs and develops^21^. There is even evidence that the survival rate of patients with PDAC has further declined^22^. According to statistics, the ratio of incidence to mortality of pancreatic cancer is 1:0.9. There are increasing concerns that PDAC may gradually rise in the ranking of cancer-related causes of death^23^. Existing biomarkers, such as *KRAS,* can predict the prognosis of PDAC, but they are not effective enough^24^. Thus, there is an urgent need to identify new and effective prediction markers. The progression of precancerous lesions to PDAC is also accelerated by the loss of several tumor suppressors^25^. IPMN is a precursor to PDAC^26^; however, there have been few prior studies evaluating the DEGs between IPMN and PDAC. Therefore, in this study, we conducted a bioinformatics analysis and identified the DEGs between IPMN and PDAC.

Based on the results of enrichment analysis, we concluded that these DEGs are closely related to the ECM and TME, which are reflected in cell adhesion, metabolism, and angiogenesis. Survival analysis is a key method for screening prognostic markers, and ROC curves can be used to evaluate this predictive power. After creating a hierarchy of hub genes and performing survival analysis, *SDC1* and *ITGA2* were finally selected as two survival-related biomarkers of PDAC from the DEGs between PDAC and IPMN.

Syndecan-1 (*SDC1*) belongs to the syndecan family^27^. As a member of cell surface transmembrane acetyl heparan sulfate proteoglycans, *SDC1* is involved in cell–cell and cell–matrix interactions, cell proliferation, and cell migration. In addition, it affects inflammation, wound-healing process, and tumor progression by controlling the above-mentioned cell functions. *SDC1*^high^ has varying significance in different kinds of cancer. *SDC1*^high^ might predict a poor prognosis of breast cancer, but a better prognosis of colorectal cancer^28,29^. To date, few studies have investigated the role of *SDC1* in PC^30^. In 2005, Juuti et al.^31^ conducted an experimental study and confirmed that the expression of interstitial *SDC1* is an independent prognostic marker of PC, whereas the expression of epithelial *SDC1* only predicts a good prognosis in resectable diseases. Notably, Yablecovitch et al.^32^ showed that serum *SDC1* levels were significantly high in patients with PDAC. Our study compared the expression of *SDC1* in PDAC and IPMN samples, and we found that the expression of *SDC1* in PDAC was higher than that in IPMN. *SDC1*^high^ indicated poor survival in patients with PDAC. The experimental results in PDAC cells confirmed that depletion of *SDC1* can significantly suppress in vitro PDAC cell proliferation, induce cell apoptosis, and impair cell migration. As confirmed by Yao et al.^33^, *SDC1* is the key medium for phagocytosis by PC macrophages. This corroborates the fact that *SDC1* regulates macrophage phagocytosis on the cell surface and promotes PDAC cell growth.

Integrin alpha 2 (*ITGA2*), a subunit of integrins, is overexpressed in malignancies and is associated with cancer progression^34^, especially reflected in the promotion of malignant behavior in tumor cell biology^35^. Nones et al.^36^ reported that *ITGA2*^high^ predicted a low survival rate in patients with PDAC. Deichmann et al.^37^ observed that immunohistochemical examination of samples from 105 patients with PDAC showed high *ITGA2* expression in 43% of patients. For patients with PDAC who had undergone resection, *ITGA2* is a biomarker that could predict their prognosis. Our results supported this conclusion. PD-L1 on the surface of tumor cells can inhibit the anti-tumor activity of CD8*T cells. Overexpression of *ITGA2* can activate the STAT3 signal pathway and upregulate the expression of PD-L1, thus promoting the invasion of malignant tumors, which are closely related to immunity^38–40^. Islam et al.^41^ used bioinformatics to analyze the prognostic role of *ITGA2* in PDAC; however, they did not perform experimental validation, making the results less credible. We not only verified the effect of *ITGA2* on the biological behavior of PC cells in vitro, but also performed a preliminary exploration of the mechanism. Our results showed that the specific mechanism by which *ITGA2*^high^ exerts its pro-pancreatic cancer effects might be closely related to the TME. Wu et al.^41^ evaluated the effects of *ITGA2* inhibitors in vivo using a KrasG12D-driven mouse model of PC. Notably, their results showed that when *ITGA2* was pharmacologically inhibited, the pre-TME was counteracted, and pancreatic injury was reversed. Zhou et al.^42^ found that *ITGA2* inhibited DNA repair in PC and consequently exerted a radio-sensitizing effect, which may be a novel perspective for the application of *ITGA2* as a target.

As a recognizable precursor of PC, the progression of IPMN appears to be markedly immune-tolerant. The immune microenvironment of IPMN is not as severe as pancreatic intraepithelial neoplasia (PanIN), even though immunity is increasingly suppressed during the transformation of IPMN to PDAC^43^. As shown in the GSEA results, the IFN-α response pathway and *KRAS* signaling pathway are the two pathways that are significantly enriched when *SDC1* is highly expressed. qRT-PCR results showed that the knockdown of *SDC1* promoted IFN-α expression, validating the conclusions obtained using GSEA. There is no direct evidence that *ITGA2* plays a role in PC metastasis; nonetheless, our experimental study showed that *ITGA2* promotes the migration of BxPC-3 and MIA-PaCa2 cells. In addition, we found that *ITGA2*^high^ signifies EMT based on the GSEA results. Subsequent experimental results confirmed that the knockdown of *ITGA2* effectively inhibited EMT. It is well known that EMT activates cancer cell metastasis, precisely because of the characteristics of mesenchymal cells that epithelial cells enhance cell movement and migration^44^. Increased expression of pro-inflammatory cytokines may be associated with response to immunotherapy and better prognosis. Furthermore, our results showed that *ITGA2* expression was positively correlated with the infiltration of multiple immune cells. Both *SDC1*^low^ and *ITGA2*^low^ were significantly enriched in pancreatic β cells, and inhibition of their expression may protect β cells from damage. We also confirmed that inhibiting *SDC1* and *ITGA2* expression makes mouse tumors smaller in vivo. In summary, targeting the *SDC1* and *ITGA2* pathways alone or in combination with immunotherapy may improve the survival of patients with PDAC.

Most IPMNs never develop into PDAC and can be safely monitored. The treatment of IPMN is sometimes extensive. The current basis for the treatment of IPMN relies on the Fukuoka guidelines, European evidence-based guidelines for cystic neoplasms of the pancreas, International Pancreatic Association guidelines, and expert opinion. Strong scientific evidence related to predicting which IPMNs are at high risk and will develop into aggressive diseases is lacking^45^. Therefore, identifying biomarkers that can predict the risk of malignant transformation remains a highly meaningful but challenging task^46^. *SDC1* and *ITGA2* are among the DEGs identified in IPMN and PDAC, and monitoring their expression is of great value in predicting whether IPMN will develop into PDAC with a poorer prognosis. Further studies are required to confirm their efficacy.

Clinical analysis demonstrated the prognostic value of *SDC1* and *ITGA2*, with high expression, predicting poor survival rates in PDAC. This predictive power was accepted, as demonstrated by the ROC curves. However, studies on prognostic biomarkers for PDAC often lack clinical validation, and our study suffers from this limitation. Nevertheless, in general, our study identified two biomarkers in silico: *SDC1* and *ITGA2*. In addition, we verified their inhibitory effect on the biological behavior of PDAC cells by knocking down their expression in vitro and conducted a preliminary exploration of their mechanisms. Although an in vivo experiment was not performed, our results support the conclusions of this study on *SDC1* and *ITGA2*. We believe that *SDC1* and *ITGA2* may also be biomarkers for detecting the conversion of IPMN to PDAC, which warrants further study. With the development of multidisciplinary cooperation and technology, the prediction of PC prognosis and the discovery of newer treatments may continue to improve. *SDC1* and *ITGA2* should be considered in strategies evaluating combinations of targeted therapy and immunotherapy.

## Conclusion

We conclude that *SDC1* and *ITGA2* are potential prognostic biomarkers for PDAC associated with IPMN. The downregulation of *SDC1* and *ITGA2* expression in PDAC may occur via a mechanism involving regulation of the IFN-α response, EMT, and immunity, which could act as potential new targets for PDAC therapy.

## Material and methods

### Ethics statement

This study was supervised by the Ethics Committee of Guang’anmen Hospital (IACUC-GAMH-2023-004).

### Data collection

The expression profiles of the sequencing datasets GSE26647, GSE63104, GSE16515, and GSE28735 of the Gene Expression Omnibus (GEO) (https://www.ncbi.nlm.nih.gov/geo/)^47^ were downloaded. They are all publicly available. The specific data information is listed in Table S2.

RNA expression and clinical data are still downloaded in the usual way^16^. The data of the Cancer Genome Atlas (TCGA) and Genotype-Tissue Expression (GTEx) databases were downloaded from the University of California at Santa Cruz Xena database (https://xenabrowser.net/datapages/). We then transformed the gene expression matrix into log_2_ (TPM + 1) values for further use^48^. The clinical information included stage, sex, age, and race^49^.

### Identification of differentially expressed genes in IPMN and PDAC samples

DEGs were filtered using the R package ‘limma’^50^. A *P*-value < 0.05 and an absolute log fold-change (FC) > 0.5 for the DEGs were defined as the cutoff. The DEGs were visualized using the R packages. The ‘complexheatmap’ package was used to generate heat maps. The ‘ggplot2’ package was used to draw volcano maps^51^.

### Enrichment analysis of DEGs

Gene Ontology (GO) enrichment analysis was used to reveal the potential biological processes of IPMN-related DEGs in PDAC. We then visualized the results using the R packages ‘Goplot’ and ‘ggplot2′^51^. Kyoto Encyclopedia of Genes and Genomes (KEGG) enrichment analysis^52,53^ was performed using the R package ‘clusterProfiler’^54,55^.

### Identification of hub genes

In the first step, the protein–protein interaction (PPI) network was constructed via the STRING database (http://string-db.org/)^56^. In step two, Cytoscape software (3.8.0) was used to visualize the PPI network. In the last step, the “CytoHubba” plugin was applied to identify significant gene clusters sorted by degree. The top ten genes were considered hub genes^57^.

### Selection of survival-related biomarkers

We identified survival-related biomarkers from the top ten hub genes through Kaplan–Meier (K–M) survival analysis. A total of 178 patients with PDAC were divided into two groups (‘High expression’ and ‘Low expression’) based on an automatically generated best cutoff value. Two important indicators were calculated: Hazard ratios (HR) with 95% confidence intervals (CI) and Cox regression *P*-values.

### Clinical analysis of survival-related biomarkers

We compared the expression of survival-related biomarkers in normal and tumor tissue samples from TCGA-PAAD and GTEx datasets^58^. We then created a receiver operating characteristic (ROC) curve using the ‘SurvivalROC’ R package^58^. We analyzed their correlation with the clinicopathological features of PC using the clinical information obtained from the TCGA-PAAD dataset.

### Molecular mechanism of survival-related biomarkers

Gene set enrichment analysis (GSEA) was performed based on the TCGA-PAAD and HALLMARK databases to investigate the biological functions and potential signaling pathways of survival-related biomarkers^59^. They were considered significantly enriched if the false discovery rate was < 0.25, *P*-value was < 0.05, and |LogFC| was > 0.2.

We analyzed the correlations between survival-related biomarkers and immune cells in PDAC tissues using TIMER (https://cistrome.shinyapps.io/timer/).

### Cell culture and treatment

The BxPC-3 and MIA-PaCa2 cell lines, derived from human PDAC, were purchased from the China Center for Type Culture Collection (CCTCC, Wuhan, China). The cells were grown in a humidified incubator containing 5% CO_2_. The complete medium contained Dulbecco’s modified Eagle’s medium (DMEM; HyClone, Cat#SH30022.01, USA), 10% certified fetal bovine serum (FBS; BI, Cat#04–001-1ACS, Israel), and 1% penicillin–streptomycin. The temperature of the incubator was set at 37 °C.

### Experimental animals

In accordance to the ARRIVE Guidelines before and during experiments, all methods were performed in accordance with the relevant guidelines and regulations, animals were also housed in compliance with institutional guidelines of Guang’anmen Hospital. Male BALB/c nude mice (4–5 weeks old, average weight 20 g) were purchased from Beijing Vital River Laboratory Animal Technology Co., Ltd. The mice were acclimatized for 1–2 weeks prior to the experiments and were housed in a pathogen-free environment with a temperature of 25 ± 2 °C, humidity of 50 ± 5%, and ad libitum access to food and water.

### siRNA transfection

siRNAs were designed and synthesized by RiboBio Co., Ltd. (Guangzhou, China). Opti-MEM (Invitrogen, Cat#31985-070, USA) and Lipofectamine™ 3000 Transfection Reagent (Invitrogen, Cat#L3000-008, USA) were used for the transfection of siRNAs, according to the manufacturer’s instructions. NC represents the negative control group.

### Quantitative real-time polymerase chain reaction

Quantitative real-time PCR (qRT-PCR) was performed to verify the interference efficiency of siRNA transfection using the ChamQ SYBR qPCR Master Mix (Vazyme, Cat#Q311-02, Nanjing, China) on an RT-PCR system (CFX96 Touch; Bio-Rad, USA) according to the manufacturer’s protocol. The gene expression was calculated using the 2^−ΔΔ*CT*^ value method, which was performed in triplicate^60^. The primers used for qRT-PCR were designed by Beijing Qingke Biotechnology Co., Ltd. (Qingdao Project Department, China). Primer sequences for genes and the experimental procedure are listed in Supplementary Table S3.

### Western blotting

Western blotting was performed as previously described^61^. The primary antibodies included those for *SDC1* (Cat#10593-1-AP, Proteintech Group, Wuhan, China), *ITGA2* (Proteintech Group, Cat#24552-1-AP). GAPDH was used for normalization. The immunoblots were examined with a visible imaging system (Bio-Rad Laboratories, Hercules, CA, USA).

### Cell proliferation assay

BxPC-3 and MIA-PaCa2 cells were seeded in 96-well plates (5 × 10^3^ cells/well). Cell Counting Kit-8 (CCK-8) solution (10-μL; Beyotime, Cat#C0038, Shanghai, China) was pipetted into each well at the indicated time points (24, 48, and 72 h). The absorbance at 450 nm was measured using a multifunctional enzyme labeling instrument (Imark-22353, Bio-Rad Laboratories). Cell viability was calculated using GraphPad Prism software. An EdU Cell Proliferation Kit with Alexa Fluor 555 (Beyotime, Cat#C0075L) was used to show the cell viability of different groups clearly.

### Cell apoptosis assay

Apoptotic cells were detected using Annexin V-FITC along with the PI solution using a flow cytometry assay. Add the corresponding reagent according to the product instructions (Annexin V-FITC apoptosis detection kit, Vazyme, Cat# A211-02). The number of cells and reagent dose were determined according to experimental conditions. Flow cytometric analysis was performed using a flow cytometer (BD AccuriC6 Plus Flow Cytometer, Franklin Lakes, NJ, USA). Photographs were taken three times randomly.

### Wound-healing assay

A wound-healing assay was used to detect the migration of BxPC-3 and MIA-PaCa2 cells. BxPC-3 and MIA-PaCa2 cells were transfected with si-SDC1, si-ITGA2, or NC in 6-well plates and cultured for 48 h until 90% confluence was reached. Wound closure was visualized at 0 and 24 h. The distance between the wound edges was calculated as previously described^62^.

### Animal experiments

The BxPC-3, MIA-PaCa2, si-SDC1-BxPC-3, si-SDC1-MIA-PaCa2, si-ITGA2-BxPC-3, and si-ITGA2-MIA-PaCa2 cell suspensions were added to a 1:3 formulation of Matrigel and PBS. Next, 5 × 10^6^ cells were subcutaneously injected into the back of the right upper limbs of nude mice. The sizes of tumors in these nude mice were measured. Tumor volume (V) was calculated using the equation V = L × W^2^/2, where L denotes long diameter and W denotes short diameter. The BxPC-3 and MIA-PaCa2 mice were then divided into two groups of six mice each, namely, the low-volume group and high-volume group. The mice in our study were terminated on day 35 and all mice were euthanised at this time point in the form of lethal doses of anaesthesia.

### Statistical analysis

Data were expressed as the mean ± SD or number of cases (%). For clinical data, the Wilcoxon test was used to test the statistical differences between multiple variables. For the categorical variables, the chi-squared test or Fisher’s exact test was used. We used R (version 4.2.1) software to conduct the statistical analyses. T-test was used in two-group comparisons to determine the variability of data obtained from in vitro experiments. For multiple groups, the one-way analysis of variance was used. Logistic regression analysis was used to determine the correlation between tumor volume and mRNA expression. GraphPad Prism (V8.2.1) was used to analyze the experimental data. Differences were considered statistically significant when the *P*-value was < 0.05.^1^

### Supplementary Information

Supplementary Information 1.

Supplementary Information 2.

## Data Availability

Article/Supplementary Material containing the original contributions is available. Contact the corresponding author for more information.
