# Supplementary material for: SDC1 and ITGA2 as novel prognostic biomarkers for PDAC related to IPMN
Source: Sci Rep. 2023 Oct 31;13:18727. doi: 10.1038/s41598-023-44646-x (PMC10618477; doi:10.1038/s41598-023-44646-x)

Figure S1. Kaplan-Meier curves.

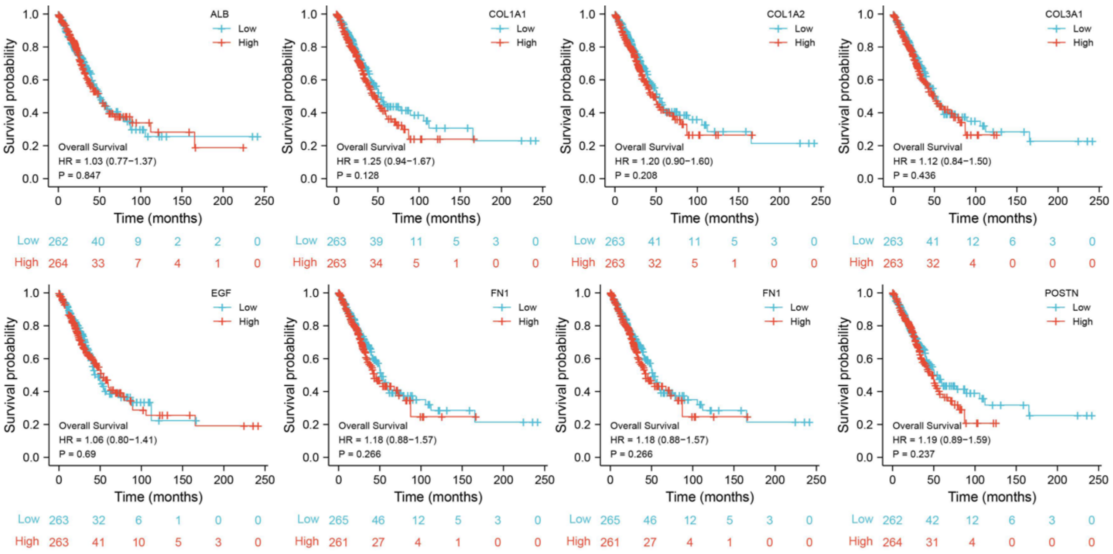

Figure S2. EdU assay.

A

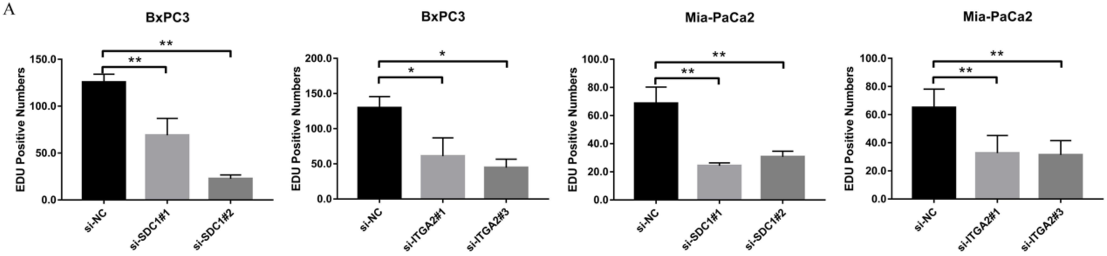

Table S1. Ten differentially expressed hub genes shared between IPMN and PC samples.

Gene name abbreviation	Full name	Degree
FN1	Fibronectin 1	57
ALB	Albumin	47
MMP9	Matrix Metallopeptidase 9	36
EGF	Epidermal Growth Factor	35
COL1A1	Collagen Type I Alpha 1 Chain	34
COL1A2	Collagen Type I Alpha 2 Chain	27
COL3A1	Collagen Type III Alpha 1 Chain	25
SDC1	Syndecan 1	25
POSTN	Periostin	24
ITGA2	Integrin Subunit Alpha 2	23

Table S2. Gene expression profile data characteristics.

Reference	PMID	Record	Tissue	Platform	Tumor	IPMN
Jury et al.[62]	22273699	GSE26647	IPMN	GPL5175 [HuEx-1_0-st] Affymetrix Human Exon 1.0 ST Array [transcript (gene) version]	-	28
Permuth-Wey et al.[63]	25607660	GSE63104	IPMN	GPL15048 Rosetta/Merck Human RSTA Custom Affymetrix 2.0 microarray [HuRSTA_2a520709.CDF]	-	23
Pei et al.[64]	19732725	GSE16515	PDAC	GPL570 [HG-U133_Plus_2] Affymetrix Human Genome U133 Plus 2.0 Array	36	-
Zhang et al.[65]	22363658	GSE28735	PDAC	GPL6244 [HuGene-1_0-st] Affymetrix Human Gene 1.0 ST Array [transcript (gene) version]	45	-

Table S3. Primer sequences for genes in qRT-PCR and experimental procedure.

	Name	Forward (5'-3')	Reverse (5'-3')
Primer sequences for genes	*SDC1*	GGAGCTCCACTCTGCTTCTC	GGGAGAGGCTGCTTCAGTTT
	ITGA2	GGGAATCAGTATTACACAACGGG	CCACAACATCTATGAGGGAAGGG
	E-cad	GCCTCCTGAAAAGAGAGTGGAAG	TGGCAGTGTCTCTCCAAATCCG
	N-cad	CCTCCAGAGTTTACTGCCATGAC	GTAGGATCTCCGCCACTGATTC
	Snail	TGCCCTCAAGATGCACATCCGA	GGGACAGGAGAAGGGCTTCTC
	Twist	GCCAGGTACATCGACTTCCTCT	TCCATCCTCCAGACCGAGAAGG
	IFN-α	AGAAGGCTCCAGCCATCTCTGT	TGCTGGTAGAGTTCGGTGCAGA
	IL-1β	CCACAGACCTTCCAGGAGAATG	GTGCAGTTCAGTGATCGTACAGG
	IL-6	AGACAGCCACTCACCTCTTCAG	TTCTGCCAGTGCCTCTTTGCTG
	TNF-α	CTCTTCTGCCTGCTGCACTTTG	ATGGGCTACAGGCTTGTCACTC
	GAPDH	CCTTCCGTGTCCCCACT	GCCTGCTTCACCACCTTC
Experimental procedure	The qRT-PCR reaction (20 μL) consisted of 10 μL 2×SYBR qPCR Master Mix, 1 μL template DNA, 0.4 μL forward and reverse primers, respectively, and 8.2 μL double-distilled deionized water. The procedure was as follows: 95 °C for 2 min; 40 cycles of 95 °C for 10 s, 60 °C for 10 s, and 95 °C for 15 s; 60 °C for 60 s; and 95 °C for 15 s.		

Table S4. Raw data for figure 8G.

SDC1:

	0 day		7 day		14 day		21 days		28 day		35 day	
**Animal counts**	**Length (mm)**	**Width (mm)**	**Length (mm)**	**Width (mm)**	**Length (mm)**	**Width (mm)**	**Length (mm)**	**Width (mm)**	**Length (mm)**	**Width (mm)**	**Length (mm)**	**Width (mm)**
1	0.00	0.00	2.32	2.27	4.61	4.25	7.4	6.25	9.21	8.34	11.96	10.67
2	0.00	0.00	2.25	2.10	3.16	2.73	6.91	5.75	7.38	5.93	8.79	7.19
3	0.00	0.00	2.29	1.64	4.36	4.04	7.68	6.57	8.54	8.04	10.92	9.83
4	0.00	0.00	2.16	2.11	3.54	3.35	6.88	5.89	8.65	7.12	9.01	7.88
5	0.00	0.00	2.21	1.59	3.28	2.71	6.17	5.83	8.77	6.87	9.52	8.59
6	0.00	0.00	2.27	1.66	3.88	2.75	7.85	7.12	9.97	8.32	11.64	10.52
7	0.00	0.00	2.39	2.22	3.75	2.69	7.26	7.14	9.28	8.18	10.79	10.61
8	0.00	0.00	2.31	1.94	4.49	4.45	7.71	7.31	8.96	8.33	10.49	8.92
9	0.00	0.00	2.14	2.12	3.35	3.81	6.78	6.04	8.31	8.02	9.69	9.48
10	0.00	0.00	2.27	1.84	4.35	3.65	7.24	6.93	9.49	8.49	11.19	9.57
11	0.00	0.00	2.22	2.07	4.23	3.13	6.56	5.16	8.12	5.42	8.71	6.69
12	0.00	0.00	2.17	1.71	3.40	3.03	6.58	5.56	7.93	5.95	9.24	7.72

ITGA2:

	0 day		7 day		14 day		21 days		28 day		35 day	
**Animal counts**	**Length (mm)**	**Width (mm)**	**Length (mm)**	**Width (mm)**	**Length (mm)**	**Width (mm)**	**Length (mm)**	**Width (mm)**	**Length (mm)**	**Width (mm)**	**Length (mm)**	**Width (mm)**
1	0.00	0.00	2.77	1.87	4.09	3.04	6.81	5.43	8.05	6.18	8.47	6.63
2	0.00	0.00	3.01	2.18	4.24	3.50	7.15	6.72	9.86	7.89	11.91	10.89
3	0.00	0.00	3.28	3.15	4.72	3.82	7.8	6.53	10.04	8.16	10.82	10.47
4	0.00	0.00	3.03	2.24	4.60	4.52	7.97	7.12	10.14	7.95	10.97	10.22
5	0.00	0.00	2.59	2.20	3.89	3.19	7.21	5.09	8.32	5.94	9.32	8.37
6	0.00	0.00	2.01	1.68	3.27	3.02	7.18	4.96	8.18	5.65	8.02	7.34
7	0.00	0.00	3.06	2.22	3.77	3.03	7.01	5.25	8.32	5.63	10.86	8.26
8	0.00	0.00	2.44	1.87	3.07	2.67	6.86	4.78	7.96	6.18	9.82	8.34
9	0.00	0.00	3.27	1.96	4.48	4.03	7.73	6.93	9.84	8.32	10.91	9.51
10	0.00	0.00	3.02	2.28	4.50	4.16	7.77	6.39	10.13	8.03	10.87	9.43
11	0.00	0.00	3.16	1.62	4.47	3.62	7.94	5.93	9.85	8.52	10.66	9.65
12	0.00	0.00	2.65	2.14	4.03	2.52	6.67	4.79	8.14	5.58	9.48	7.79

Table S5. Raw data for figure 8H.

BxPC3:

si NC group	0 day		7 day		14 day		21 days		28 day		35 day	
Animal Numbers	Length (mm)	Width (mm)	Length (mm)	Width (mm)	Length (mm)	Width (mm)	Length (mm)	Width (mm)	Length (mm)	Width (mm)	Length (mm)	Width (mm)
1	0.00	0.00	3.35	2.44	5.08	4.05	8.67	6.72	10.42	8.06	12.57	10.64
2	0.00	0.00	3.02	2.34	5.24	3.62	7.45	6.74	10.16	8.65	11.83	10.01
3	0.00	0.00	3.71	2.93	5.21	3.72	8.22	6.14	11.89	8.70	12.41	10.53
4	0.00	0.00	3.38	3.19	5.52	4.09	7.79	5.46	10.25	8.75	11.48	11.02
5	0.00	0.00	3.32	2.82	5.12	3.74	8.23	6.76	10.18	9.39	12.72	10.78
6	0.00	0.00	3.18	2.98	5.06	4.26	7.74	6.11	9.55	8.66	11.20	10.25
si SDC1+ITGA2 group	0 day		7 day		14 day		21 days		28 day		35 day	
Animal Numbers	Length (mm)	Width (mm)	Length (mm)	Width (mm)	Length (mm)	Width (mm)	Length (mm)	Width (mm)	Length (mm)	Width (mm)	Length (mm)	Width (mm)
1	0.00	0.00	2.42	2.11	4.06	2.73	6.12	4.44	7.29	6.09	8.93	7.13
2	0.00	0.00	2.28	2.03	3.64	2.31	5.95	4.06	6.78	6.24	6.99	6.57
3	0.00	0.00	2.31	1.46	3.32	2.08	5.5	4.12	7.71	6.48	8.58	7.94
4	0.00	0.00	2.47	2.17	2.96	2.59	5.02	3.36	7.59	6.81	8.97	7.99
5	0.00	0.00	2.96	1.72	3.69	2.76	5.37	4.06	7.76	6.50	9.65	7.83
6	0.00	0.00	2.56	1.26	4.06	2.47	5.08	3.97	6.62	6.04	7.59	6.70

MIA-PaCa2:

si NC group	0 day		7 day		14 day		21 days		28 day		35 day	
Animal Numbers	Length (mm)	Width (mm)	Length (mm)	Width (mm)	Length (mm)	Width (mm)	Length (mm)	Width (mm)	Length (mm)	Width (mm)	Length (mm)	Width (mm)
1	0.00	0.00	2.54	1.92	4.75	3.05	7.03	5.45	9.85	7.46	11.70	9.94
2	0.00	0.00	3.10	2.66	5.41	4.11	6.57	5.88	8.77	8.01	10.69	8.90
3	0.00	0.00	2.37	2.28	4.98	4.01	7.41	6.18	9.61	8.68	11.13	10.26
4	0.00	0.00	2.92	2.43	5.02	4.03	6.95	5.52	10.07	7.99	11.85	10.58
5	0.00	0.00	2.19	2.12	4.82	3.78	7.42	6.49	9.43	7.72	10.48	9.68
6	0.00	0.00	2.54	1.85	5.07	3.58	6.97	6.15	8.61	7.43	10.83	9.59
si SDC1+ITGA2 group	0 day		7 day		14 day		21 days		28 day		35 day	
Animal Numbers	Length (mm)	Width (mm)	Length (mm)	Width (mm)	Length (mm)	Width (mm)	Length (mm)	Width (mm)	Length (mm)	Width (mm)	Length (mm)	Width (mm)
1	0.00	0.00	2.10	1.43	3.83	2.82	5.52	3.89	7.12	5.13	9.12	7.65
2	0.00	0.00	2.13	1.08	3.52	2.58	4.7	4.24	7.59	6.07	8.49	7.28
3	0.00	0.00	1.75	1.27	3.64	2.18	4.57	3.32	7.16	5.37	8.68	6.63
4	0.00	0.00	1.61	1.39	3.76	2.94	4.47	4.55	8.36	6.59	9.52	7.79
5	0.00	0.00	1.96	1.14	3.34	2.77	4.52	3.52	7.29	5.27	8.96	7.93
6	0.00	0.00	1.79	1.32	3.22	2.13	4.99	4.58	7.45	4.66	9.14	7.68

The results of Western Blot.

(A) BxPC-3:

si-SDC1 and GAPDH

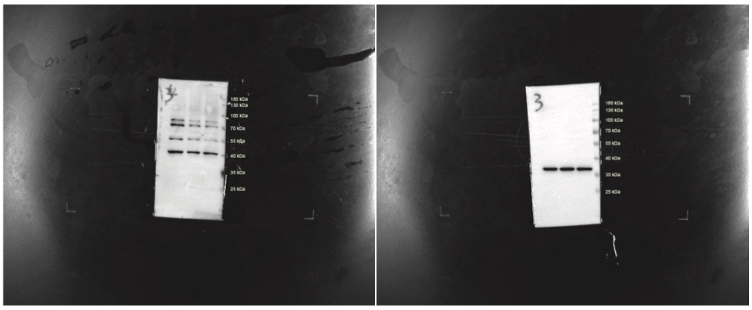

si-ITGA2 and GAPDH

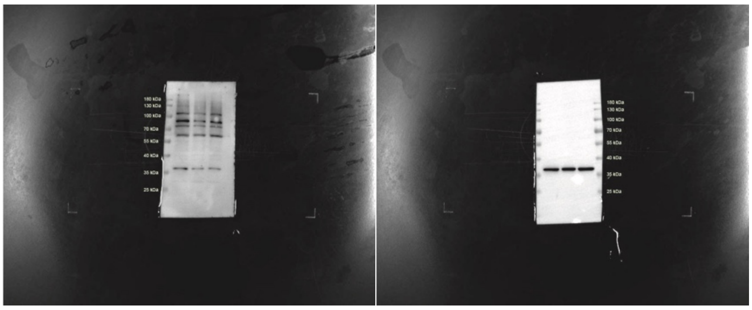

(B) MIA-PaCa2:

si-SDC1 and GAPDH

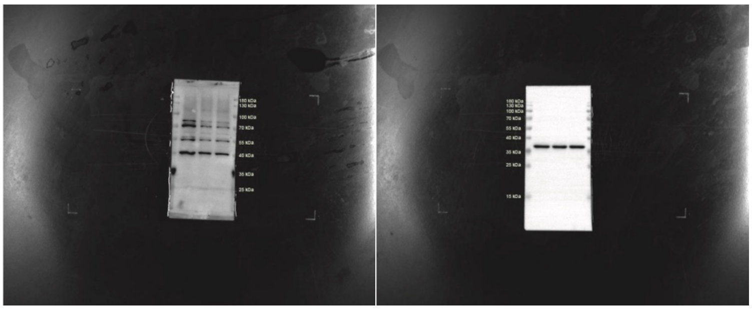

si-ITGA2 and GAPDH

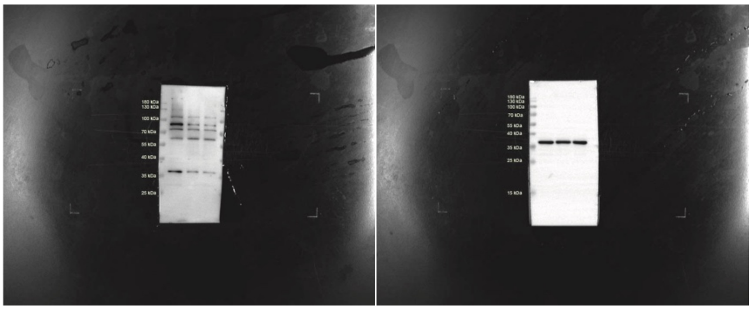

Supplement: Supplementary file 1 — Supplementary Information 1. [file 41598_2023_44646_MOESM1_ESM.pdf]
